# Enhanced production of amyrin in *Yarrowia lipolytica* using a combinatorial protein and metabolic engineering approach

**DOI:** 10.1186/s12934-022-01915-0

**Published:** 2022-09-09

**Authors:** Jing Kong, Lin Miao, Zhihui Lu, Shuhui Wang, Baixiang Zhao, Cuiying Zhang, Dongguang Xiao, Desmond Teo, Susanna Su Jan Leong, Adison Wong, Aiqun Yu

**Affiliations:** 1 State Key Laboratory of Food Nutrition and Safety, Key Laboratory of Industrial Fermentation Microbiology of the Ministry of Education, Tianjin Key Laboratory of Industrial Microbiology, College of Biotechnology, Tianjin University of Science and Technology, No.29 the 13th Street TEDA, Tianjin, 300457 People’s Republic of China; 2 Food, Chemical and Biotechnology Cluster, Singapore Institute of Technology, Singapore, 138683 Singapore

**Keywords:** *Y. lipolytica*, Triterpenoid, Amyrin, Metabolic engineering, Protein engineering

## Abstract

**Background:**

Amyrin is an important triterpenoid and precursor to a wide range of cosmetic, pharmaceutical and nutraceutical products. In this study, we metabolically engineered the oleaginous yeast, *Yarrowia lipolytica* to produce α- and β-amyrin on simple sugar and waste cooking oil.

**Results:**

We first validated the in vivo enzymatic activity of a multi-functional amyrin synthase (CrMAS) from *Catharanthus roseus*, by expressing its codon-optimized gene in *Y. lipolytica* and assayed for amyrins. To increase yield, prevailing genes in the mevalonate pathway, namely *HMG1*, *ERG20*, *ERG9* and *ERG1*, were overexpressed singly and in combination to direct flux towards amyrin biosynthesis. By means of a semi-rational protein engineering approach, we augmented the catalytic activity of CrMAS and attained ~ 10-folds higher production level on glucose. When applied together, protein engineering with enhanced precursor supplies resulted in more than 20-folds increase in total amyrins. We also investigated the effects of different fermentation conditions in flask cultures, including temperature, volumetric oxygen mass transfer coefficient and carbon source types. The optimized fermentation condition attained titers of at least 100 mg/L α-amyrin and 20 mg/L β-amyrin.

**Conclusions:**

The design workflow demonstrated herein is simple and remarkably effective in amplifying triterpenoid biosynthesis in the yeast *Y. lipolytica*.

**Supplementary Information:**

The online version contains supplementary material available at 10.1186/s12934-022-01915-0.

## Introduction

Terpenoids, i.e., terpenes and their functionalized derivatives, are isoprene-based natural products with fundamental roles in plant metabolism. Due to their structural diversity and abundance, terpenoids have found important applications in the flavors, fragrances and pharmaceutical industries. Notably, terpenoid compounds have been shown to display antimicrobial, anti-inflammatory, antidiabetic, anticancer and analgesic properties and are being pursued as active ingredients or adjuvants in drug formulations [1, 2]. Terpenoid compounds are further classified according to their number of isoprene units, including mono-, sesqui-, di-, sester-, triterpenoids and etc. [3].

α- and β-amyrins are representative members of triterpenoids that differ only in the placement of its methyl group on the triterpene skeleton. Importantly, amyrins are used as precursors for the downstream biosynthesis of other valuable bioactive compounds, including avenacine, centellosides, ginsenosides and glycyrrhizin [4, 5]. In addition, amyrins have been shown to exhibit anti-inflammatory, antidiabetic and anticancer effects in several studies [6, 7]. Traditionally, amyrins are obtained by either chemical synthesis or phytoextraction from the resins, leaves and stem barks of flowering plants. These methods typically suffer from low efficiency, high energy consumption and generate a large amount of organic waste, hence adding to the overall production cost.

Complemented by advances in gene synthesis, expression engineering and pathway discovery, biologists have refined metabolic engineering principles to develop microbial cell factories that can synthesize non-native, plant natural products [8, 9]. Numerous terpenoid compounds, including limonene, bisabolol, isoprene and squalene, have been produced by fermentation technology [10, 11]. While reviewing literatures on triterpenoid biosynthesis in yeasts, we came across two pioneering studies of enabling amyrin biosynthesis in the Baker’s yeast, *Saccharomyces cereivisae*. In the first study, the expression of codon-optimized β-amyrin synthase from *Glycyrrhiza glabra*, with concurrent overexpression of truncated hydroxymethylglutaryl-CoA reductase tHMG1, farnesyl diphosphate (FPP) synthase ERG20, squalene synthase ERG9 and squalene monooxygenase ERG1 on synthetic promoters, achieved 105 mg/L production titers in a 5 L bioreactor under glucose fed-batch fermentation [12]. A higher β-amyrin titer, up to 280 mg/L, was recorded in a subsequent study with augmented acetyl-CoA supply in the engineered *S. cerevisiae* [13]. In the second study, the introduction of codon-optimized multifunctional amyrin synthase from *Malas domestica*, coupled with overexpression of *ERG20*, *ERG9* and *ERG1* genes in the mevalonate (MVA) pathway, resulted in α-amyrin accumulation at ~ 12 mg/L in flask cultures [14].

**Fig. 1:**
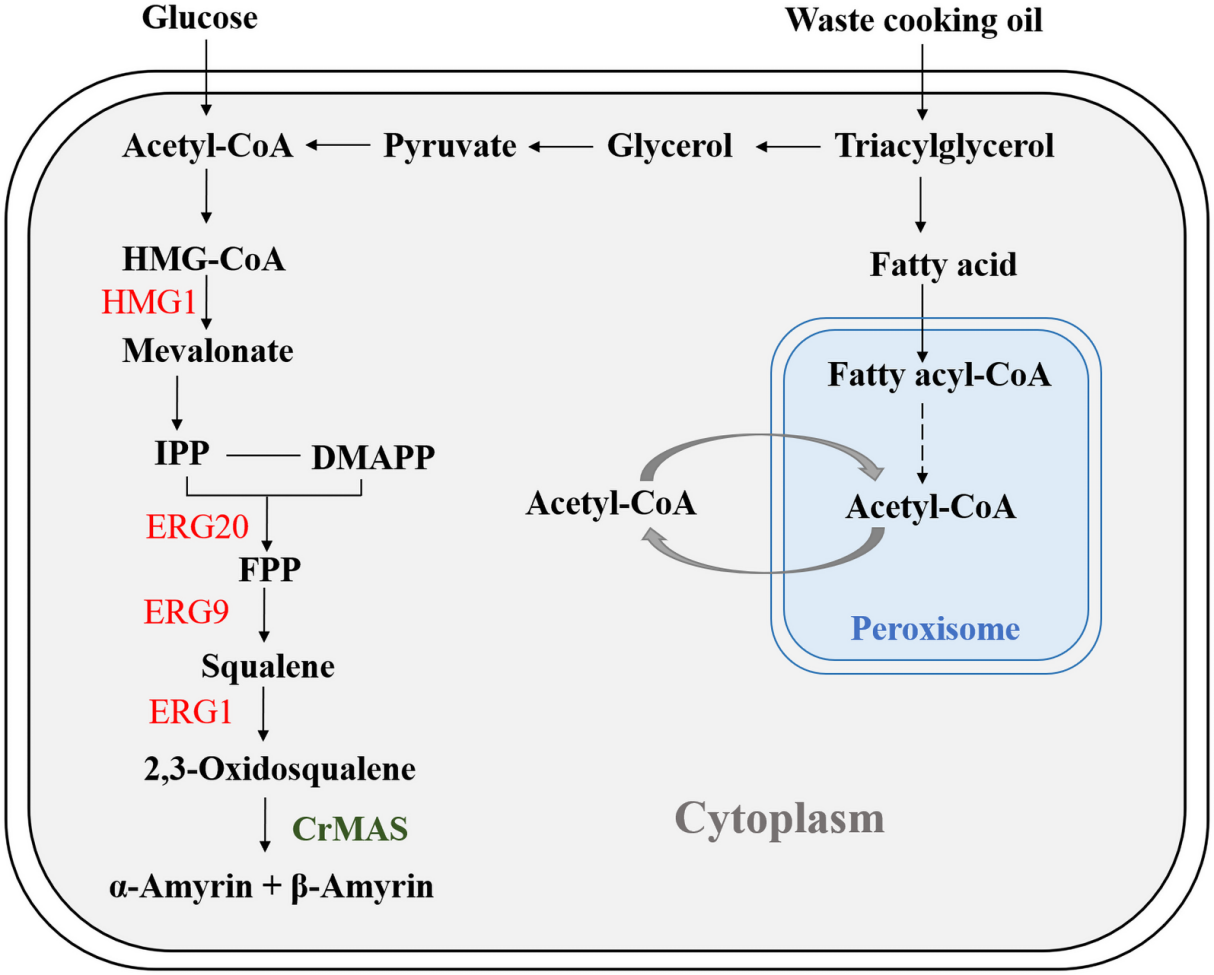
Biosynthesis of α- and β-amyrins in the engineered *Y. lipolytica*. Extracellular carbon sources were assimilated and converted into α- and β amyrins via the mevalonate (MVA) pathway. Metabolites are identified in black. Endogenous and foreign genes that were up-regulated are listed in red and green, respectively. HMG1: hydroxymethylglutaryl-CoA reductase; ERG20: farnesyl diphosphate synthase; ERG9: squalene synthase; ERG1: squalene monooxygenase; CrMAS: *C. roseus* multifunctional amyrin synthase

Theoretically, the unconventional yeast *Yarrowia lipolytica* may be more favorable, as an engineered cell factory, than the conventional yeast *S. cerevisiae* in producing triterpenoids. Besides its metabolic plasticity and generally regarded as safe (GRAS) status, as an oleaginous yeast, *Y. lipolytica* has higher abundance of intracellular acetyl-CoA precursors that can be used in the downstream of MVA biosynthetic pathway (Fig. 1) [15, 16]. This is not the case for *S. cerevisiae* in which the pyruvate decarboxylation pathway supplying cytosolic acetyl-CoA is strongly competed by the ethanol fermentation [17]. More importantly, *Y. lipolytica* can metabolize a variety of carbon substrates for growth, including waste cooking oil (WCO) and mixed food waste hydrolysate [18, 19]. This permits the valorization of waste streams and reduces the overall cost of production.

The MVA pathway in fungi branches differently from that of plants at the 2,3-oxidosqualene node. In fungi, the metabolite is channeled towards ergosterol biosynthesis. In plants, however, the metabolite is utilized in both phytosterols and triterpene biosynthesis, which are both critical for survival [20]. Oxidosqualene cyclases (OSCs) is a class of plant enzymes that convert 2,3-oxidosqualene into the various triterpene and sterol scaffolds [21]. Being a membrane protein, it is fundamentally challenging to obtain sufficient quantities of purified protein for characterization from its natural sources. The heterologous expression of OSCs in yeast is thus confined to studying the biochemistry of these enzymes in vivo [22–24]. In a recent study, the expression of a multifunctional amyrin synthase from *Catharanthus roseus* (CrMAS) in *S. cerevisiae* was reported to produce ~ 40 mg/L α-amyrin and ~ 10 mg/L β-amyrin in glucose-grown shake-flask cultures [25]. Naturally occurring enzymes may function suboptimally or not at all when introduced into a heterologous host, in part due to differences in the host or chemical context [26]. For this reason, protein engineering is often considered in metabolic engineering projects for strain optimization [27].

In this study, we report on the engineering of *Y. lipolytica* to produce mixed amyrins. We first corroborated the compatibility of CrMAS in *Y. lipolytica* by expressing a codon-optimized version of the gene and detected trace amounts of α- and β-amyrin in flask cultures. To increase yield, prevailing genes in the MVA pathway, namely *HMG1*, *ERG20*, *ERG9* and *ERG1*, were overexpressed singly and in combination to direct flux towards amyrin biosynthesis. Following a semi-rational protein engineering approach, we augmented the catalytic activity of CrMAS and attained ~ 10-folds higher production level than the control strain on glucose. When applied together, protein engineering with enhanced precursor supplies resulted in more than 20-folds increase in total amyrins. We also investigated the effects of different fermentation conditions in flask cultures, including temperature, volumetric oxygen mass transfer coefficient and carbon source types. The optimized fermentation condition attained titers of 100 mg/L α-amyrin and 20 mg/L β-amyrin. At the time of writing, this represents the highest reported mixed amyrin titers in the engineered *Y. lipolytica* cell factory [28, 29]. Importantly, our study validates the case to consider protein design in the metabolic engineering of triterpenoid biosynthesis in the yeast *Y. lipolytica*.

## Results and discussion

### Heterologous expression of a codon-optimized CrMAS enabled amyrin production in *Y. lipolytica*

**Fig. 2:**
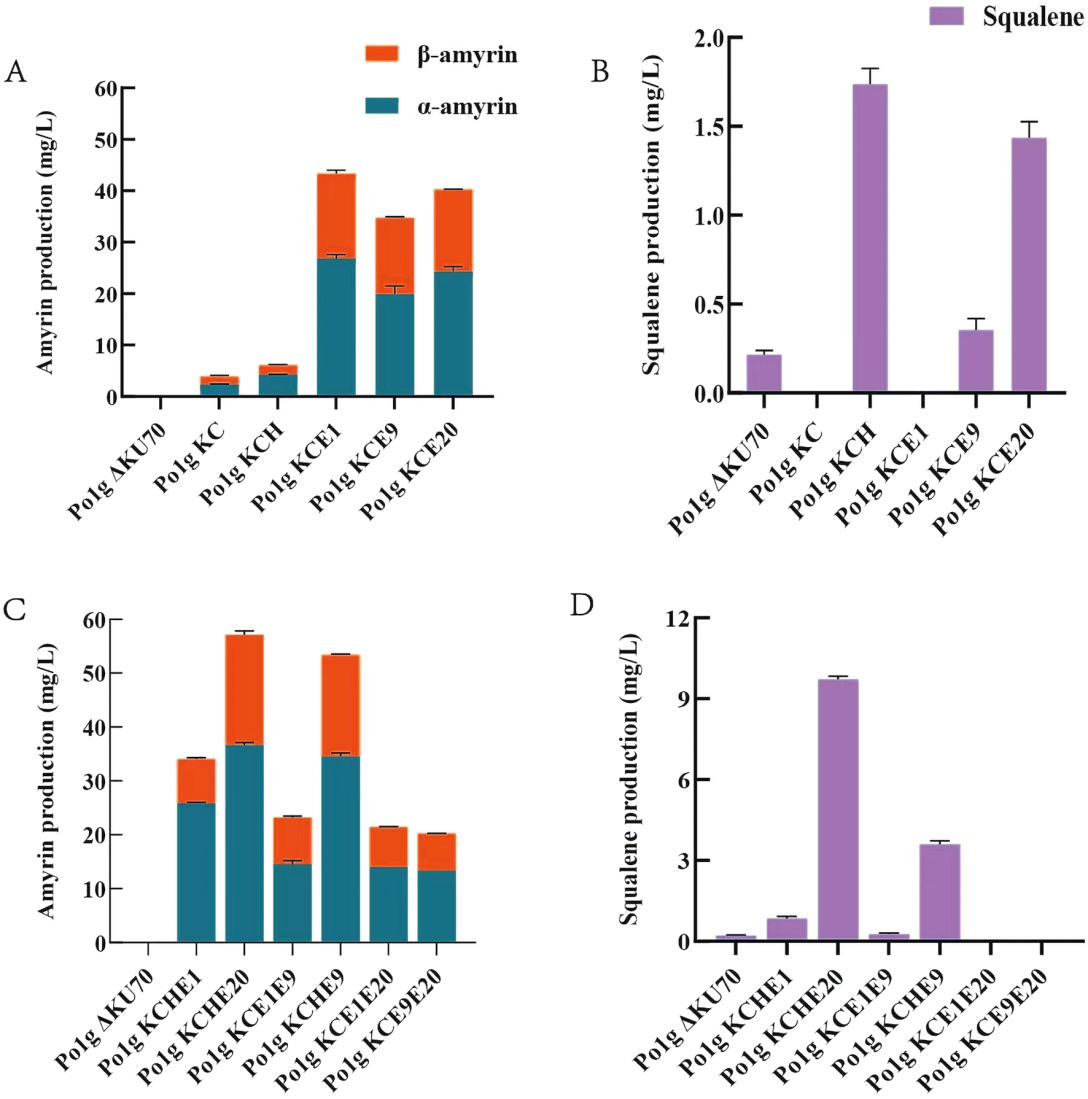
Triterpenoid titers in the engineered *Y. lipolytica* in YPD. Prevailing genes in the MVA pathway, namely *HMG1*, *ERG20*, *ERG9* and *ERG1*, were overexpressed singly and in combination. **A** Amyrin titers with single gene overexpression. **B** Squalene titers with single gene overexpression. **C** Amyrin titers with multiple gene overexpression. **D** Squalene titers with multiple gene overexpression. Control strain: Po1g ΔKU70. The codon-optimized *CrMAS* gene was used in all the engineered strains. Amyrins were quantified after 5 days of cultivation in shake flasks. Each engineered strain was named based on the genes that were overexpressed. The codon-optimized *CrMAS* gene: C; *HMG1* gene: H; *ERG1* gene: E1; *ERG9* gene: E9; *ERG20* gene: E20. For example, the Po1g KCHE20 strain refers to *Y. lipolytica* Po1g ΔKU70 overexpressing *CrMAS*, *HMG1* and *ERG20*. All values presented are the mean of three biological replicates ± standard deviation

Emerging interests in plant triterpenoid biosynthesis have resulted in the isolation and characterization of a repertoire of OSCs. The multifunctional amyrin synthase from the pink periwinkle plant *C. roseus*, CrMAS, was previously reported to produce both α- and β-amyrin from 2,3-oxidosqualene when expressed in *S. cerevisiae* [25]. Taking insight from this, we overexpressed *CrMAS* gene codon optimized for *Y. lipolytica* with the synthetic promoter pHP4D, resulting in the creation of strain Po1g KC. As a growth-phase dependent promoter, pHP4D permits gene expression mostly at the onset of stationary phase [30]. This facilitates efficient resource allocation for cell growth and bioproduction. After 5 days of fermentation in YPD, we detected trace amount of mixed amyrins which are comprised of 2.39 mg/L α-amyrin and 1.42 mg/L β-amyrin (Fig. 2A, Additional file 1: Table S1). Amyrin was not detected in the control strain Po1g ΔKU70 that was without CrMAS. Interestingly, the opposite was true for squalene, the precursor of 2,3-oxidosqualene (Fig. 2B, Additional file 1: Figures S1, S2), hence confirming that amyrin synthesis could only be due to CrMAS protein. No growth defect was detected, indicating that overexpression of this enzyme did not result in cellular toxicity (Additional file 1: Figure S3).

### Increased precursor availability led to higher amyrin titers in the engineered *Y. lipolytica*

In the endogenous MVA pathway of yeast strains, carbon flux leading to 2,3-oxidosqualene, which is the precursor for α- and β-amyrins, can be enhanced by overexpression of prevailing genes *HMG1*, *ER20*, *ERG9* and *ERG1* (Fig. 1) [12]. HMG1 enzyme, one of two isoforms of 3-hydroxy-3-methylglutaryl-CoA (HMG-CoA) reductase in yeasts, is often identified as the rate-limiting enzyme in this pathway [31, 32]. In several studies on the engineering of terpenoid biosynthesis in *S. cerevisiae*, a truncated version of HMG1 is overexpressed in place of the original enzyme. This is because feedback inhibition by enzyme degradation can be decoupled by removing its N-terminal ubiquitination signal [33]. In *Y. lipolytica*, however, it is ambiguous whether the catalytic activity of tHMG1 is better than the original enzyme [34].

To increase amyrin titers, therefore, endogenous genes listed in Fig. 1 were each overexpressed with the pHP4D promoter (together with codon-optimized CrMAS) and integrated into the chromosome of *Y. lipolytica* Po1g ΔKU70 thereafter. This resulted in the creation of the strains Po1g KCH, Po1g KCE1, Po1g KCE9 and Po1g KCE20, which displayed up-regulated expression of *HMG1*, *ERG1*, *ERG9* and *ERG20* (besides *CrMAS*) genes, respectively. Accordingly, all the engineered strains with overexpression of the identified genes produced more total amyrins when compared with the control strain Po1g KC expressing only CrMAS (Fig. 2A). Three strains with the singly overexpressed genes, Po1g KCE1, Po1g KCE9 and Po1g KCE20, showed between 8.8 and 11.1-folds increase in amyrin titers (Additional file 1: Table S1). Unexpectedly, strain Po1g KCH, intended for high expression of the known rate-limiting step in the MVA pathway, showed only very modest increase in amyrin titers, < 1.6-folds (Fig. 2B). However, intracellular squalene accumulated to higher amount in Po1g KCH, thus agreeing with previous studies that HMG1 was rate-limiting in certain contexts. Squalene was absent in both the ERG1 up-regulated and Po1g KC control, implying that the intermediate metabolite was fully consumed (Fig. 2B). To identify synergistic genes in the MVA pathway, we developed a library of strains with up-regulated expressions of prevailing genes in pair-wise permutations. Fortuitously, strains Po1g KCHE9 and Po1g KCHE20 produced more mixed amyrins than when *HMG1*, *ERG9* or *ERG20* were singly overexpressed, attaining impressive titers of 52.90 mg/L and 56.40 mg/L, respectively (Fig. 2C). Other engineered strains did not benefit from dual gene overexpression, with a trivial amount of squalene or not at all (Fig. 2D).

### Alanine substitution at target amino acid residue enhanced CrMAS bioconversion of 2,3-oxidosqualene to amyrin

**Fig. 3:**
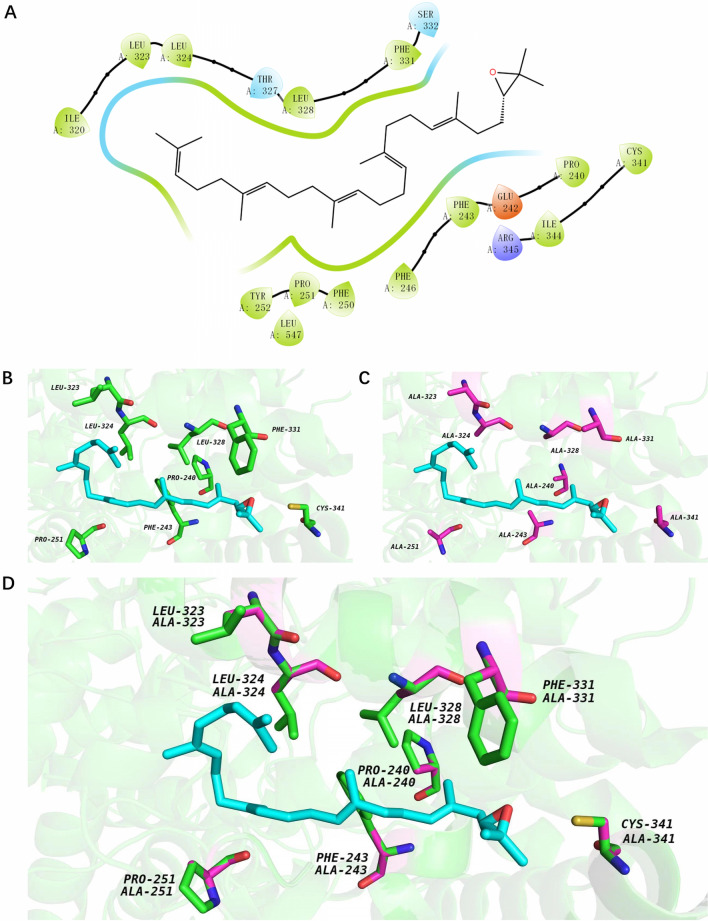
Close-up perspective of the predicted CrMAS substrate binding cluster, Site 1, visualized using PyMOL. **A** 2-D representation of the substrate access tunnel, of wild-type CrMAS. Hydrophobic amino acids in green, polar amino acids in blue, positively charged amino acids in purple, negatively charged amino acid residues in red. 3-D models of the substrate access tunnel of **B** wild-type CrMAS, **C** mutated CrMAS, and **D** superimposed of both. Site-directed mutagenesis were performed to change the eight target amino acids to alanine, as indicated in pink. Molecular structure of 2,3-oxidosqualene is displayed in cyan

In the engineered pathway leading to amyrin biosynthesis, CrMAS is the only non-endogenous protein involved that is distantly associated with fungi. We thus posited that remodeling of wild type CrMAS for better stability and specific activity may increase the overall amyrin titers. To this end, we applied a semi-rational protein engineering workflow that involved molecular docking simulation to predict probable substrate binding clusters and the subsequent mutagenesis of target amino acid residues to alanine. This approach has the advantage to create smaller and smarter libraries that is guided by the sequence and structural information of the analyzed protein. Molecular coupling of 2,3-oxidosqualene substrate to the amyrin synthase was modelled using the Schrödinger software package, in which a lower docking score denoted a higher probability of substrate binding. A total of five promising substrate binding pockets, each comprising of a cluster of interacting amino acid residues, were identified (Additional file 1: Figure S4). Our simulation results also suggest that the hypothetical substrate binding pocket, herein designated as Site 1 (Fig. 3A–D and Additional file 1: Figure S4), had the strongest binding potential with 2,3-oxidosqualene (Table 1).

**Table 1:** The docking scores of 2,3-oxidosqualene in predicted binding pockets of CrMAS protein

Predicted Binding Pockets	Score
Site 1 (PHE331, LEU328, LEU324, LEU323, PHE243, PRO240, CYS341, PRO251)	− 4.621
Site 2 (TYR692, ALA429, ILE428)	− 3.829
Site 3 (TYR314, TYR315, MET668, VAL546, PRO542)	− 2.837
Site 4 (ALA696, TYR752, PRO700)	− 2.407
Site 5 (PRO456, VAL452)	− 3.052

A more negative score represents higher probability of substrate binding event within the cluster of amino acid residues

**Fig. 4:**
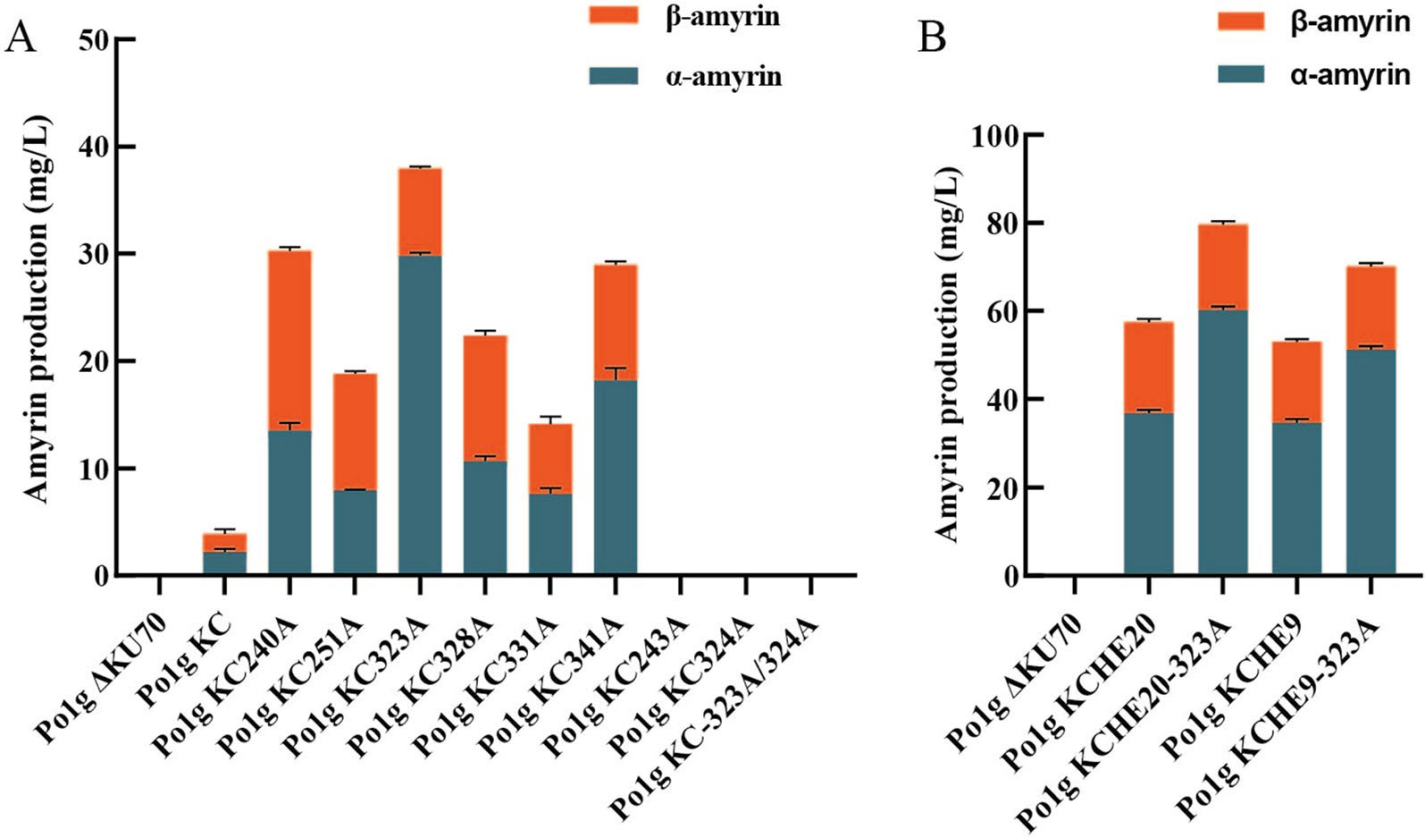
Amyrin titers in the engineered *Y. lipolytica* with mutated CrMAS in YPD. **A** Effects of different alanine point mutation on the enzymatic activity of CrMAS. **B** Expression of the best performing mutant CrMAS L323A in strains metabolically engineered with enhanced precursor supplies. Control strains: Po1g ΔKU70 and Po1g KC. Amyrins were quantified after 5 days of cultivation in shake flasks. All values presented are the mean of three biological replicates ± standard deviation

Substituting key amino acid residues with alanine has been shown to enhance the catalytic activities of various proteins [35, 36]; hence, site-directed mutagenesis were performed to convert the eight candidate residues of CrMAS into alanine (Table 1: P240, F243, P251, L323, L324, L328, F331 and C341). Yeast strains expressing the eight mutants were denoted as Po1g KC-240A, Po1g KC-243A, Po1g KC-251A, Po1g KC-323A, Po1g KC-324A, Po1g KC-328A, Po1g KC-331A and Po1g KC-341A, respectively. Remarkably, six out of the eight mutants showed improved catalysis, with 3-folds rise in total amyrin titers over the wild type (Fig. 4A, Additional file 1: Table S1). Strain Po1g KC-323A with the CrMAS L323A mutation attained the highest α-amyrin titer at 29.60 mg/L, an increase of 12-folds, while strain Po1g KC-240A with the P240A mutation has the highest β-amyrin titer at 16.46 mg/L, an increase in 11-folds. A comparison of CrMAS 3D-homology models before and after alanine substitutions suggested that there was significant relief in steric hindrance within the substrate access tunnel in the six enhanced CrMAS mutants (Fig. 3B and C). Mutation of residues lining the access tunnel is known to alter enzyme properties including substrate specificity, enantioselectivity and product release [37]; faster product release reduces enzyme occupancy, and thus may explicate the overall increase in catalytic activity. Interestingly, both F243A and L324A mutants with no measurable catalytic activity were shown to be in almost direct contact with the skeletal structure of 2,3-oxidosqualene substrate before the alanine substitutions, suggesting that both may be conserved residues (Fig. 3B). Point mutations at these two residues could have implicated substrate and enzyme interaction, thereby resulting in a loss of function. In line with this theory, the P240A mutant remained in contact with the substrate even after mutation, thus the enzyme’s activity was retained and even enhanced after the change.

### Combination of protein engineering with precursor flux channeling further increased amyrin titers in the engineered *Y. lipolytica*

To further enhance amyrin production in *Y. lipolytica*, we combined both protein and metabolic engineering strategies discussed above and created strains Po1g KCHE9-323A and Po1g KCHE20-323A. This was accomplished by means of integrative plasmids pYLCH9-323A and pYLCH20-323A, which harbored pHP4D-mediated overexpression of CrMAS L323A, HMG1, and either ERG9 or ERG20. Remarkably, strain Po1g KCHE20-323A achieved the highest amyrin titers among all others developed in this study, with a record titer of 59.7 mg/L α-amyrin and 19.9 mg/L β-amyrin (Fig. 4B, Additional file 1: Table S1). This embodied a 140% increase over the Po1g KCHE20 strain with overexpression of wild type CrMAS, HMG1 and ERG20. In all, the results are consistent with our hypothesis that a combination of protein engineering and precursor flux channeling would be more effective than when each method was used alone.

### Amyrin production by the engineered *Y. lipolytica* in flask cultures

**Fig. 5:**
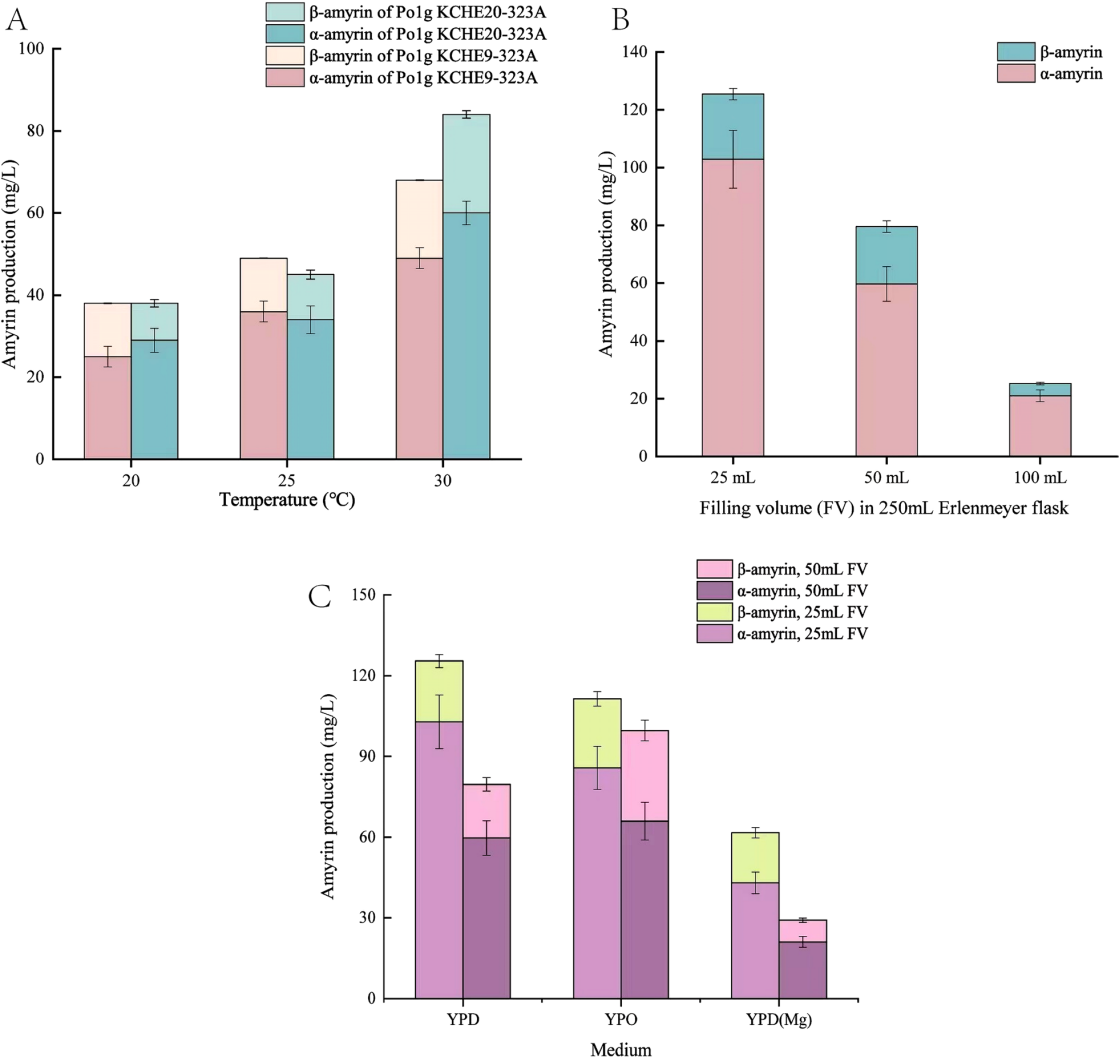
Production titers of *Y. lipolytica* amyrin-high producers under different cultivation conditions. **A** Different temperatures for strains Po1g KCHE9-323A and Po1g KCHE20-323A in YPD. **B** Different filling volumes, FV. Strain Po1g KCHE20-323A in YPD. FV of 100 mL, 50 mL and 25 mL correspond to k_L_a of 16 h^−1^, 39 h^−1^ and 84 h^−1^, respectively. **C** Different media formulation. YPD: yeast-peptone-glucose; YPD(Mg): YPD supplemented with magnesium sulphate; YPO: yeast-peptone-waste cooking oil. Amyrins were quantified after 5 days of cultivation in shake flasks. All values presented are the mean of three biological replicates ± standard deviation

When employing microbial cell factories to produce platform chemicals, it is critical that high product titers can be attained consistently in consideration of the extracellular environment. Optimum oxygen concentrations and temperatures are essential to balance yeast cell growth and enzyme activities, while the choice of media ingredients directly affects the economic feasibility of the process. First, to study the effects of culture temperature on production titer, we measured the total amyrin titer obtained on YPD media for strains Po1g KCHE9-323A and Po1g KCHE20-323A grown at 20 °C, 25 °C and 30 °C after 5 days of shake flask cultivation. These two strains were chosen because they were identified to be the best amyrin producers among all strains created in this study (Fig. 4B, Additional file 1: Table S1). Our results show that amyrin production was most favored at 30 °C for both engineered *Yarrowia* strains, with nearly twice the amount of mixed amyrins obtained when fermentation was conducted at 20 °C (Fig. 5A). Given that Po1g KCHE20-323A produced the most amyrins at 30 °C, we next evaluated the strain’s production capability in different aeration environment. Specifically, Po1g KCHE20-323A was grown in 250 mL Erlenmeyer flasks each with different filling volumes (FV) of YPD media. Then, using established modelling correlation, we associated the obtained amyrin titers to different estimated k_L_a values in our experimental set-ups (Fig. 5B); 16 h^−1^ (100 mL FV), 39 h^−1^ (50 mL FV) and 84 h^−1^ (25 mL FV) (Additional file 1: Figure S5 and Additional file 2). As expected, amyrin production was directly proportional to maximum k_L_a, in the shake flask cultures (and hence inversely proportional to FV). Using glucose as the carbon source, at least 125 mg/L of mixed amyrins titer was attained on YPD, consisting of nearly 103 mg/L α-amyrin and 22 mg/L β-amyrin (Fig. 5B, Additional file 1: Table S2).

In previous studies, we had established that Mg^2+^ supplementation to YPD media can increase the titer of limonene and bisabolene product in engineered *Y. lipolytica* without affecting its growth. This is in part due to the ion’s ancillary role as cofactors in key enzymatic reactions [38–40]. To test if this would also be the case for amyrin production, we compared the titers obtain from strain Po1g KCHE20-323A in YPD(Mg) and YPD after 5 days of shake flask cultivation. Results from Fig. 5C, however, indicated otherwise, with 35% lower amyrin titers observed in the engineered *Yarrowia* cultures with Mg^2+^ supplementation. Conservatively, around 190 million metric tons of WCO are generated each year [41], thus raising concerns of waste management. Fermentative valorization of WCO can be an effective approach to both reduce waste as well as provide sustainable feedstock for the biomanufacturing industry. To test the feasibility of using WCO for amyrin production, we formulated the YPO media, consisting of yeast extract, peptone and WCO, and repeated shake flask fermentation in 25 mL and 50 mL filling volume set-ups. As shown in Fig. 5C, using WCO as the primary carbon source for *Yarrowia* fermentation was workable and achieved ~ 89% of mixed amyrin titers on glucose. This corresponded to a total amyrin titer of at least 110 mg/L, with nearly 85 mg/L α-amyrin and 25 mg/L β-amyrin (Fig. 5C, Additional file 1: Table S2). Interestingly, the use of WCO was also able to overcome the k_L_a limits. In fact, there were more amyrins produced on WCO than that of glucose in 50 mL FV set-up which corresponded to kLa of 39 h^−1^. At larger bioreactor scale, fermentative bioprocess that is operated at lower k_L_a may incur lower production cost. Nevertheless, a comprehensive technoeconomic analysis should be performed to ascertain if the benefits of lower production cost can offset the drawback of reduced production titer.

## Conclusion

In this study, we applied a combinatorial engineering strategy, consisting of both protein and metabolic engineering to achieve substantial amyrin biosynthesis in *Y. lipolytica*. We also leveraged on *Y. lipolytica*’s intrinsic ability to utilize WCO as feedstock and tested a small scale bioprocess for circular and cost-effective production of triterpenoids [18]. At the optimal temperature of ~ 30 °C and k_L_a of ~ 84 h^−1^, amyrin titers of at least 125 mg/L (103 mg/L α-amyrin and 22 mg/L β-amyrin) and 110 mg/L (85 mg/L α-amyrin and 25 mg/L β-amyrin) were attained in glucose and WCO shake flask cultures, respectively (Additional file 1: Table S2). To date, this represents the highest amount of amyrins produced in this oleaginous yeast. While the registered titers are still a moonshot from being commercially viable, at least 20 g/L in laboratory scale [42], the present study serves to benefit the scientific community through communication of the design framework and knowledge gained.

Amyrin biosynthesis in *Y. lipolytica* strains expressing wild type CrMAS was conspicuously improved when HMG1 was overexpressed alongside either ERG20 or ERG9. The two strains, namely Po1g KCHE20 and KCHE9, also exhibited higher intracellular accumulation of squalene precursors, thus indicating that the precursor was generated at rates faster than it was used by ERG1 and CrMAS. Comparatively, single overexpression of the identified pathway genes was less effective in raising amyrin yields. Surprisingly, the double overexpression of ERG20 and ERG9 without HMG1, did not result in higher amyrin yields when either of the genes was overexpressed singly; instead, squalene was completely mopped out below the detection limit. This may likely be related to the feedback regulation of FPP on the MVA pathway, at least in part as a positive signal for the degradation of HMG1 [43]. Our results indicate that simply overexpressing all genes in the MVA pathway leading to the target product, amyrins in this case, may not always be favorable. Given the multifaceted nature of MVA pathway regulation, measurements of the intracellular abundance of each metabolite and the activity of related enzymes are crucial in resolving intrinsic bottlenecks. One way that can be realized is by using refactored promoters of differing strengths to drive the expression of prevailing genes in the pathway, hence decoupling their expression from native feedback mechanisms. Following, surface response methodology can be applied to analyze metabolomics data and guide the fine-tuning of bioconversion in vivo. Another momentous aspect of this study is the validation of a semi-rational protein engineering method, guided by molecular docking simulation, to enhance enzymatic activity. Our results affirm that substitution of the target amino acids to the non-polar and less sterically hindered alanine can significantly boost biocatalysis and improve amyrin yields [44]. Moving forward, the development of structure-based, machine learning algorithm that considers the evolutionary trade-off between enzyme stability and activity may guide the creation of superior enzyme variants [45].

## Materials and methods

### Strains, plasmids and cultivation media

Plasmids and strains used in this study are listed in Tables 2 and 3, respectively. Plasmid pYLEX1 was used as the integrative vector for *Y. lipolytica* in this study. *Y. lipolytica* Polg ΔKU70 was used as the host for all genetic modifications with chromosomal expression constructs introduced via engineered pYLEX1 plasmids; the KU70-deficient mutant exhibits a higher rate of homologous recombination events and is more genetically tractable. *Y. lipolytica* strains were routinely cultivated in yeast peptone dextrose media (YPD) comprising of 10 g/L yeast extract, 20 g/L peptone and 20 g/L glucose unless otherwise specified. *Escherichia coli* DH5α was used for cloning and plasmid amplification. *E. coli* transformants were grown in Luria–Bertani ampicillin-selective media (LBA) comprising of 5 g/L yeast extract, 10 g/L peptone, 10 g/L NaCl and 100 μg/mL of ampicillin.

**Table 2:** List of plasmids employed in this study

Plasmid	Description	Source
pYLEX1	*Y. lipolytica* integrative plasmid, pHP4D-tXPR2, leu2, Ap^r^	^40^
pUCCrMAS	codon-optimized (naturally occurring) crmas, Apr	Beijing Genomics Institute
pYLCrMAS	pHP4D-crmas-tXPR2, leu2, Ap^r^	This study
pYLE1	pHP4D-erg1-tXPR2, leu2, Ap^r^	This study
pYLE9	pHP4D-erg9-tXPR2, leu2, Ap^r^	This study
pYLE20	pHP4D-erg20-tXPR2, leu2, Ap^r^	This study
pYLH	pHP4D-hmg1-tXPR2, leu2, Ap^r^	This study
pYLC-240A	pHP4D-crmasP240A-tXPR2, leu2, Ap^r^	This study
pYLC-243A	pHP4D-crmasF243A-tXPR2, leu2, Ap^r^	This study
pYLC-251A	pHP4D-crmasP251A-tXPR2, leu2, Ap^r^	This study
pYLC-323A	pHP4D-crmasL323A-tXPR2, leu2, Ap^r^	This study
pYLC-324A	pHP4D-crmasL324A-tXPR2, leu2, Ap^r^	This study
pYLC-323A/324A	pHP4D-crmasL323A/L324A-tXPR2, leu2, Ap^r^	This study
pYLC-328A	pHP4D-crmasL328A-tXPR2, leu2, Ap^r^	This study
pYLC-331A	pHP4D-crmasF331A-tXPR2, leu2, Ap^r^	This study
pYLC-341A	pHP4D-crmasC341A-tXPR2, leu2, Ap^r^	This study
pYLCE1	pHP4D-crmas-tXPR2, pHP4D-erg1-tXPR2, leu2, Ap^r^	This study
pYLCE9	pHP4D-crmas-tXPR2, pHP4D-erg9-tXPR2, leu2, Ap^r^	This study
pYLCE20	pHP4D-crmas-tXPR2, pHP4D-erg20-tXPR2, leu2, Ap^r^	This study
pYLCH	pHP4D-crmas-tXPR2, pHP4D-hmg1-tXPR2, leu2, Ap^r^	This study
pYLCHE1	pHP4D-crmas-tXPR2, pHP4D-hmg1-tXPR2, pHP4D-erg1-tXPR2, leu2, Ap^r^	This study
pYLCHE9	pHP4D-crmas-tXPR2, pHP4D-hmg1-tXPR2, pHP4D-erg9-tXPR2, leu2, Ap^r^	This study
pYLCHE20	pHP4D-crmas-tXPR2, pHP4D-hmg1-tXPR2, pHP4D-erg20-tXPR2, leu2, Ap^r^	This study
pYLCE1E9	pHP4D-crmas-tXPR2, pHP4D-erg1-tXPR2, pHP4D-erg9-tXPR2, leu2, Ap^r^	This study
pYLCE1E20	pHP4D-crmas-tXPR2, pHP4D-erg1-tXPR2, pHP4D-erg20-tXPR2, leu2, leu2, Ap^r^	This study
pYLCE9E20	pHP4D-crmas-tXPR2, pHP4D-erg9-tXPR2, pHP4D-erg20-tXPR2, leu2, leu2, Ap^r^	This study
pYLCHE9-323A	pHP4D-crmasL323A-tXPR2, pHP4D-hmg1-tXPR2, pHP4D-erg9-tXPR2, leu2, Ap^r^	This study
pYLCHE20-323A	pHP4D-crmasL323A-tXPR2, pHP4D-hmg1-tXPR2, pHP4D-erg20-tXPR2, leu2, Ap^r^	This study

**Table 3:** Strains used in this study

Strains	Description	Source
*E. coli*		
DH5α	For cloning and plasmid amplification purposes; wild type	Lab-owned
*Y. lipolytica*		
Po1g ΔKU70	MATA, xpr2-332, leu2-270, ku70-, ura3-302::URA3, Axp-2; wild type	[48]
Po1g KC	MATA, xpr2-332, leu2-270, ku70-, ura3-302::URA3, Axp-2, codon-optimized crmas	This study
Po1g KCE1	MATA, xpr2-332, leu2-270, ku70-, ura3-302::URA3, Axp-2, codon-optimized crmas, erg1	This study
Po1g KCE9	MATA, xpr2-332, leu2-270, ku70-, ura3-302::URA3, Axp-2, codon-optimized crmas, erg9	This study
Po1g KCE20	MATA, xpr2-332, leu2-270, ku70-, ura3-302::URA3, Axp-2, codon-optimized crmas, erg20	This study
Po1g KCH	MATA, xpr2-332, leu2-270, ku70-, ura3-302::URA3, Axp-2, codon-optimized crmas, hmg1	This study
Po1g KCHE1	MATA, xpr2-332, leu2-270, ku70-, ura3-302::URA3, Axp-2, codon-optimized crmas, HMG1, erg1	This study
Po1g KCHE9	MATA, xpr2-332, leu2-270, ku70-, ura3-302::URA3, Axp-2, codon-optimized crmas, HMG1, erg9	This study
Po1g KCHE20	MATA, xpr2-332, leu2-270, ku70-, ura3-302::URA3, Axp-2, codon-optimized crmas, HMG1, erg20	This study
Po1g KCE1E9	MATA, xpr2-332, leu2-270, ku70-, ura3-302::URA3, Axp-2, codon-optimized crmas, erg1, erg9	This study
Po1g KCE1E20	MATA, xpr2-332, leu2-270, ku70-, ura3-302::URA3, Axp-2, codon-optimized crmas, erg1, erg20	This study
Po1g KCE9E20	MATA, xpr2-332, leu2-270, ku70-, ura3-302::URA3, Axp-2, codon-optimized crmas, erg9, erg20	This study
Po1g KCE1	MATA, xpr2-332, leu2-270, ku70-, ura3-302::URA3, Axp-2, codon-optimized crmas, erg1	This study
Po1g KC-240A	MATA, xpr2-332, leu2-270, ku70-, ura3-302::URA3, Axp-2, crmasP240A	This study
Po1g KC-243A	MATA, xpr2-332, leu2-270, ku70-, ura3-302::URA3, Axp-2, crmasF243A	This study
Po1g KC-251A	MATA, xpr2-332, leu2-270, ku70-, ura3-302::URA3, Axp-2, crmasP251A	This study
Po1g KC-323A	MATA, xpr2-332, leu2-270, ku70-, ura3-302::URA3, Axp-2, crmasL323A	This study
Po1g KC-324A	MATA, xpr2-332, leu2-270, ku70-, ura3-302::URA3, Axp-2, crmasL324A	This study
Po1g KC-323A/324A	MATA, xpr2-332, leu2-270, ku70-, ura3-302::URA3, Axp-2, crmasL323A/L324A	This study
Po1g KC-328A	MATA, xpr2-332, leu2-270, ku70-, ura3-302::URA3, Axp-2, crmasL328A	This study
Po1g KC-331A	MATA, xpr2-332, leu2-270, ku70-, ura3-302::URA3, Axp-2, crmasF331A	This study
Po1g KC-341A	MATA, xpr2-332, leu2-270, ku70-, ura3-302::URA3, Axp-2, crmasC341A	This study
Po1g KCHE9-323A	MATA, xpr2-332, leu2-270, ku70-, ura3-302::URA3, Axp-2, crmasL323A, hmg1, erg9	This study
Po1g KCHE20-323A	MATA, xpr2-332, leu2-270, ku70-, ura3-302::URA3, Axp-2, crmasL323A, hmg1, erg20	This study

### Plasmid construction

Amyrin synthase gene from *C. roseus* (*CrMAS*, GenBank: JN991165) was codon-optimized and synthesized by Beijing Genomics Institute (Shenzhen, China). The gene was then cloned into the pYLEX1 vector using the *Pml* I (New England Biolab, Ipswich, MA, USA) restriction site to generate pYLCrMAS (Additional file 1: Figure S6). *HMG1*, *ERG1*, *ERG9* and *ERG20* genes were natively cloned from *Y. lipolytica* chromosomal DNA. Primers used are presented in Additional file 1: Table S3.

### Site-directed mutagenesis

Site-directed mutagenesis were performed with KOD-Plus-Mutagenesis Kit (Toyobo Biotech, Shanghai, China) as per the manufacturer’s instructions. Briefly, pYLEX1-CrMAS was used as template and subjected to inverse PCR with primers for alanine point mutations. The resultant PCR products were digested with *Dpn* I (New England Biolab, Ipswich, MA, USA) to eliminate the background plasmid before being re-circularized by a reaction mix comprising of T4 polynucleotide kinase and ligase (New England Biolab, Ipswich, MA, USA). The ligated plasmid products were subsequently transformed into *E. coli* DH5α and verified by DNA sequencing. Primers used for site-directed mutagenesis are presented in Additional file 1: Table S4.

### Yeast transformation

Integrative plasmids were chemically transformed into *Y. lipolytica* Po1g ΔKU70 using a modified lithium acetate transformation method. The precise protocols for preparing Po1g ΔKU70 competent cells and mediating plasmid uptake are provided in the Additional Methods in Additional file 1. Yeast transformants were selected on leucine-deficient plates and confirmed by colony PCR.

### Homology modelling

Protein homology modelling and molecular docking were performed by MedChem Express (New Jersey, USA). Briefly, the Schrödinger's Protein Preparation Wizard software was used to construct a predicted, energy minimized 3D homology model of CrMAS enzyme from its raw protein databank file [46]. The Schrödinger’s Sitemap module was further employed to identify and rank potential substrate-binding pockets as shown in Table 1; a more negative score represents higher possibility of CrMAS interacting with the cognate substrate 2,3-oxidosqualene. A total of 5 docking sites were identified, each comprising of a cluster of amino acid residues located within 2.4 Å distance of the substrate access tunnel. Visualization and superimposing of the protein structures were performed using Schrödinger's PyMOL.

### GC–MS analysis

To determine squalene and amyrin, 10 mL of *Yarrowia* cultures were sampled and centrifuged for 10 min at 6000 × g. The cell pellets obtained were resuspended in 2 mL hexane and vortexed with quartz sand for 20 min. Solvent-extracted metabolites were analyzed by GC/MS (Shimadzu QP-2010 Ultra, Kyoto, Japan) equipped with a HP-5 M column (0.25 mm × 30 m × 0.25 μm) from Agilent (Santa Clara, CA, USA). The injection temperature was 280 °C. The GC oven temperature was set to 100 °C for 3 min after injection, followed by a 10 °C/min ramp up to the final temperature of 280 °C, and held for 20 min. Full mass spectra were generated for metabolite identification by scanning the m/z range of 50–500. Helium flow rate was set at 1.0 mL/min, and the sample injection volume was 1 μL. Metabolite retention times and mass spectra were compared with analytical grade chemical standards for compound identification.

### Flask fermentation

Starter cultures were first prepared by inoculating single colonies of the engineered *Y. lipolytica* strains in 5 mL YPD media and grown for 24 h in a shaking incubator set at 30 °C and 250 rpm. The cultures were transferred into 50 mL of YPD(Mg) (containing 2% peptone, 2% dextrose, 1% yeast extract, with or without 0.2% MgSO_4_·7H_2_O) in 250 mL unbaffled Erlenmeyer flasks to achieve an initial cell density of OD_600_ ~ 0.1. Flask fermentations were conducted in a uniform shaking incubator environment, set at 30 °C and 250 rpm and harvested after 5 days. For the fermentation of strain Po1g CHE20-323A in WCO, a YPO medium containing WCO (1% yeast extract, 2% peptone, 1.18% WCO and 0.2% tween-80 contained) was used. The amount of carbon from WCO in YPO was the same as that provided by glucose in YPD. For the fermentation of strain Po1g CHE20-323A in different filling volume, the volume of media used were 25 mL, 50 mL and 100 mL. The volumetric oxygen mass transfer coefficient for shake flask cultures with different filling volume, k_L_a in h^−1^, was determined using an established empirical correlation [47]. Detail calculations are presented in the additional excel file (Additional file 2).

## Supplementary Information

**Additional file 1.** Additional figures and tables.

**Additional file 2.** Correlation of volumetric oxygen mass transfer coefficient to amyrin titers.

## Data Availability

Not applicable.
