# Supplementary material for: Enhanced production of amyrin in Yarrowia lipolytica using a combinatorial protein and metabolic engineering approach
Source: Microb Cell Fact. 2022 Sep 9;21:186. doi: 10.1186/s12934-022-01915-0 (PMC9463779; doi:10.1186/s12934-022-01915-0)
Supplement: Supplementary file 1 — Additional file 1. Additional figures and tables. [file 12934_2022_1915_MOESM1_ESM.docx]

**ADDITIONAL INFORMATION**

**Enhanced production of amyrin in *Yarrowia lipolytica* using a combinatorial protein and metabolic engineering approach**

Jing Kong^a^, Lin Miao^a^, Zhihui Lu^a^, Shuhui Wang^a^, Baixiang Zhao^a^, Cuiying Zhang^a^, Dongguang Xiao^a^, Desmond Teo^b^, Susanna Su Jan Leong^b^, Adison Wong^b**^, Aiqun Yu^a*^

^a^State Key Laboratory of Food Nutrition and Safety, Key Laboratory of Industrial Fermentation Microbiology of the Ministry of Education, Tianjin Key Laboratory of Industrial Microbiology, College of Biotechnology, Tianjin University of Science and Technology, No.29 the 13th Street TEDA, Tianjin 300457, PR China.

^b^Food, Chemical and Biotechnology Cluster, Singapore Institute of Technology, Singapore 138683, Singapore.

**Co-corresponding author: Dr. Adison Wong, [Assistant Professor], [Singapore Institute of Technology], E-mail: adison.wong@singaporetech.edu.sg, Tel: +65 65921626

*Corresponding author: Dr. Aiqun Yu, [Professor], [Tianjin University of Science and Technology], E-mail: yuaiqun@tust.edu.cn, Tel: +86 22 60602723

**ADDITIONAL FILE 1: TABLES**

**Additional file 1: Table S1.** Numerical data of amyrin titers in Figures 2 and 4.

| ***Yarrowia* strains** | **α-Amyrin (mg/L)** | **β-Amyrin (mg/L)** | **Total amyrins (mg/L)** | **Squalene (mg/L)** |
| --- | --- | --- | --- | --- |
| Po1g △KU70 | Not detected | N.D | N.D | 0.23 |
| Po1g KC | 2.39 | 1.42 | 3.81 | N.D |
| Po1g KCH | 4.27 | 1.78 | 6.05 | 1.68 |
| Po1g KCE1 | 26.26 | 16.11 | 42.37 | N.D |
| Po1g KCE9 | 18.82 | 14.68 | 33.50 | 0.31 |
| Po1g KCE20 | 23.69 | 15.93 | 39.62 | 1.37 |
| Po1g KCHE1 | 25.77 | 8.37 | 34.14 | 0.80 |
| Po1g KCHE20 | 36.30 | 20.10 | 56.40 | 9.64 |
| Po1g KCE1E9 | 14.02 | 8.50 | 22.52 | 0.23 |
| Po1g KCHE9 | 34.05 | 18.85 | 52.90 | 3.52 |
| Po1g KCE1E20 | 14.01 | 7.48 | 21.49 | N.D |
| Po1g KCE9E20 | 13.31 | 6.93 | 20.24 | N.D |
| * CrMAS site-directed mutants complemented with enhanced precursor supplies | | | | |
| Po1g KCHE20-323A | 59.69 | 19.90 | 79.59 | Not measured |
| Po1g KCHE9-323A | 50.57 | 18.43 | 69.00 | N.M |
| * CrMAS site-directed mutants | | | | |
| Po1g KC-240A | 13.02 | 16.46 | 29.48 | N.M |
| Po1g KC-251A | 7.92 | 10.63 | 18.55 | N.M |
| Po1g KC-323A | 29.60 | 8.28 | 37.88 | N.M |
| Po1g KC-328A | 10.35 | 11.38 | 21.73 | N.M |
| Po1g KC-331A | 7.21 | 6.02 | 13.23 | N.M |
| Po1g KC-341A | 17.38 | 10.55 | 27.92 | N.M |
| Po1g KC-243A | N.D | N.D | N.D | N.M |
| Po1g KC-324A | N.D | N.D | N.D | N.M |
| Po1g KC-323A/324A | N.D | N.D | N.D | N.M |

**Additional file 1: Table S2.** Numerical data of amyrin titers in Figure 5.

| ***Yarrowia* strains, fermentation temperature** | **α-Amyrin** | **β-Amyrin** | **Total amyrins (mg/L)** |
| --- | --- | --- | --- |
| Po1g KCHE9-323A, 20℃ | 25.10 | 13.57 | 38.67 |
| Po1g KCHE9-323A, 25℃ | 36.60 | 13.38 | 49.99 |
| Po1g KCHE9-323A, 30℃ | 50.57 | 18.43 | 69.00 |
| Po1g KCHE20-323A, 20℃ | 29.17 | 9.46 | 38.63 |
| Po1g KCHE20-323A, 25℃ | 34.55 | 11.34 | 45.89 |
| Po1g KCHE20-323A, 30℃ | 59.69 | 19.9 | 79.59 |
| * Fermentation experiments below were performed at 30^o^C | | | |
| Po1g KCHE20-323A, FV 25mL, YPD | 102.86 | 22.56 | 125.42 |
| Po1g KCHE20-323A, FV 50mL, YPD | 59.69 | 19.90 | 79.59 |
| Po1g KCHE20-323A, FV 100mL, YPD | 21.01 | 4.23 | 25.24 |
| Po1g KCHE20-323A, FV 25mL, YPD(Mg) | 43.64 | 18.66 | 62.29 |
| Po1g KCHE20-323A, FV 50mL, YPD(Mg) | 21.07 | 8.09 | 29.16 |
| Po1g KCHE20-323A, 25mL, YPO | 85.74 | 25.65 | 111.39 |
| Po1g KCHE20-323A, 50mL, YPO | 65.96 | 33.63 | 99.59 |

**Additional file 1: Table S3.** List of primers used for constructs assembly.

| Applications | Primer | Sequence |
| --- | --- | --- |
| Amplify *CrMAS* gene from pYLCrMAS DNA, forward primer. | CrMAS-F | ACAACCACACACATCCACGTGATGTGGAAGCTGAAGATCGCC |
| Amplify *CrMAS* gene from pYLCrMAS DNA, reverse primer. | CrMAS-R | TTAGTTTCGGGTTCCCACGTGCAGGGCCTTGGTGGGCCA |
| Amplify *HMG1* gene from *Y. lipolytica* genomic DNA, forward primer. | HMG1-F | ACAACCACACACATCCACAATGCTACAAGCAGCTATTGGAAAG |
| Amplify *HMG1* gene from *Y. lipolytica* genomic DNA, reverse primer. | HMG1-R | GGGACAGGCCATGGAGGTACCCTATGACCGTATGCAAATATTCGAA |
| Amplify *ERG1* gene from *Y. lipolytica* genomic DNA, forward primer. | ERG1-F | ACAACCACACACATCCACAATGGTCACCCAACAGTCTGCA |
| Amplify *ERG1* gene from *Y. lipolytica* genomic DNA, reverse primer. | ERG1-R | TTAGTTTCGGGTTCCCACCTAAGTCAGCTCGCTCCAAATGT |
| Amplify *ERG9* gene from *Y. lipolytica* genomic DNA, forward primer. | ERG9-F | ACAACCACACACATCCACAATGGGAAAACTCATCGAACTGC |
| Amplify *ERG9* gene from *Y. lipolytica* genomic DNA, reverse primer. | ERG9-R | TTAGTTTCGGGTTCCCACCTAATCTCTCAGAGGAAACATCTTAGAGTC |
| Amplify *ERG20* gene from *Y. lipolytica* genomic DNA, forward primer. | ERG20-F | ACAACCACACACATCCACAATGTCCAAGGCGAAATTCG |
| Amplify *ERG20* gene from *Y. lipolytica* genomic DNA, reverse primer. | ERG20-R | TTAGTTTCGGGTTCCCACCTACTTCTGTCGCTTGTAAATCTTGG |
| Amplify the second gene expression cassette, forward primer. | E1-F | CCATCCAGCCTCGCGTCGAGGTTGAGGCCGTTGAGCA |
| Amplify the second gene expression cassette, reverse primer. | E1-R | ACGTCTTGCTGGCGTTCGGATAAGCTGTCAAACATGAGAATTCG |
| Amplify the second or third gene expression cassette, forward primer. | E9-F | CGCATAAGGGAGAGCGTCGACGATAAGCTGTCAAACATGAGAATTCG |
| Amplify the second or third gene expression cassette, reverse primer. | E9-R | CTCTCAAGGGCATCGGTCGACCCGTTGAGCACCGCCGCC |
| Amplify the second or third gene expression cassette, forward primer. | A-F | ATCCTGCGATGCAGATCCGGAGCCCAGTAGTAGGTTG |
| Amplify the second or third gene expression cassette, reverse primer. | A-R | AACCTACTACTGGGCTGCTGTCAAACATGAGAATTCG |
| Gene validation, forward primer. | PYL-F | CCTCGATCCGGCATGCACTGATCACG |
| Gene validation, reverse primer. | PYL-R | TAGGCAACAGCGTTGGGAGAGCCCTTGAGG |
| Gene validation, forward primer. | DJY-F | AATCGCCGTGACGATCAGC |
| Gene validation, reverse primer. | DJY-R | CTGTCGCTTGCGGTATTCGG |
| Gene validation, forward primer. | DSG-F | GTCCTACGAGTTGCATGAT |
| Gene validation, reverse primer. | DSG-R | CTGTCGCTTGCGGTATTCGG |
| Gene validation, forward primer. | T-F | AGCGTTAATGTCTGGCTTCT |
| Gene validation, reverse primer. | T-R | CTCGACCTGAATGGAAGCCG |
| Gene validation, forward primer. | 9-R | GGAAACATCTTAGAGTCGA |
| Gene validation, reverse primer. | 20-R | TCTTCTTGATGTCGCCAAC |

**Additional file 1: Table S4.** List of primers used for site-directed mutagenesis.

| Applications | Primer | Sequence |
| --- | --- | --- |
| PRO240 locus reverse amplification, forward primer. | 240-F | GCACCCGAGTTCTGGCTGTTTCCCTCTTTCTTCC |
| PRO240 locus reverse amplification, reverse primer. | 240-R | CAGAGGATTGCATCCGTCCCAGTCATACACGC |
| PHE243 locus reverse amplification, forward primer. | 243-F | GCCTGGCTGTTTCCCTCTTTCTTCCCCTACCAC |
| PHE243 locus reverse amplification, reverse primer. | 243-R | CTCGGGAGGCAGAGGATTGCATCCGT |
| PRO251 locus reverse amplification, forward primer. | 251-F | GCCTACCACCCCGCCAAGATGTGGTGC |
| PRO251 locus reverse amplification, reverse primer. | 251-R | GAAGAAAGAGGGAAACAGCCAGAACTCGGGAGGC |
| LEU323 locus reverse amplification, forward primer. | 323-F | GCCCTGTGGGACACCCTGAACTACTTCTCTGAGCC |
| LEU323 locus reverse amplification, reverse primer. | 323-R | GTCCTGGATGAAAGAGTGGGGGTAATACAGGTCGT |
| LEU324 locus reverse amplification, forward primer. | 324-F | GCCTGGGACACCCTGAACTACTTCTCTGAGCCCGT |
| LEU324 locus reverse amplification, reverse primer. | 324-R | CAGGTCCTGGATGAAAGAGTGGGGGTAATACAGG |
| LEU323/LEU324 locus reverse amplification, forward primer. | 3234-F | GCGGCGTGGGACACCCTGAACTACTTCTCTGAGCCCG |
| LEU328 locus reverse amplification, forward primer. | 328-F | GCCAACTACTTCTCTGAGCCCGTGATGCGACGA |
| LEU328 locus reverse amplification, reverse primer. | 328-R | GGTGTCCCACAGCAGGTCCTGGATGAAAGAG |
| PHE331 locus reverse amplification, forward primer. | 331-F | GCCTCTGAGCCCGTGATGCGACGATGG |
| PHE331 locus reverse amplification, reverse primer. | 331-R | GTAGTTCAGGGTGTCCCACAGCAGGTCCT |
| ALA341 locus reverse amplification, forward primer. | 341-F | GCCAATAAGATCCGAGAGAAGGCCATGCGAAAGTGC |
| ALA341 locus reverse amplification, reverse primer. | 341-R | GGGCCATCGTCGCATCACGGGCT |
| Sequencing validation forward primer. | AJS-F | CAAGAAGGAGCTGGTGCGA |

**ADDITIONAL FIGURES**


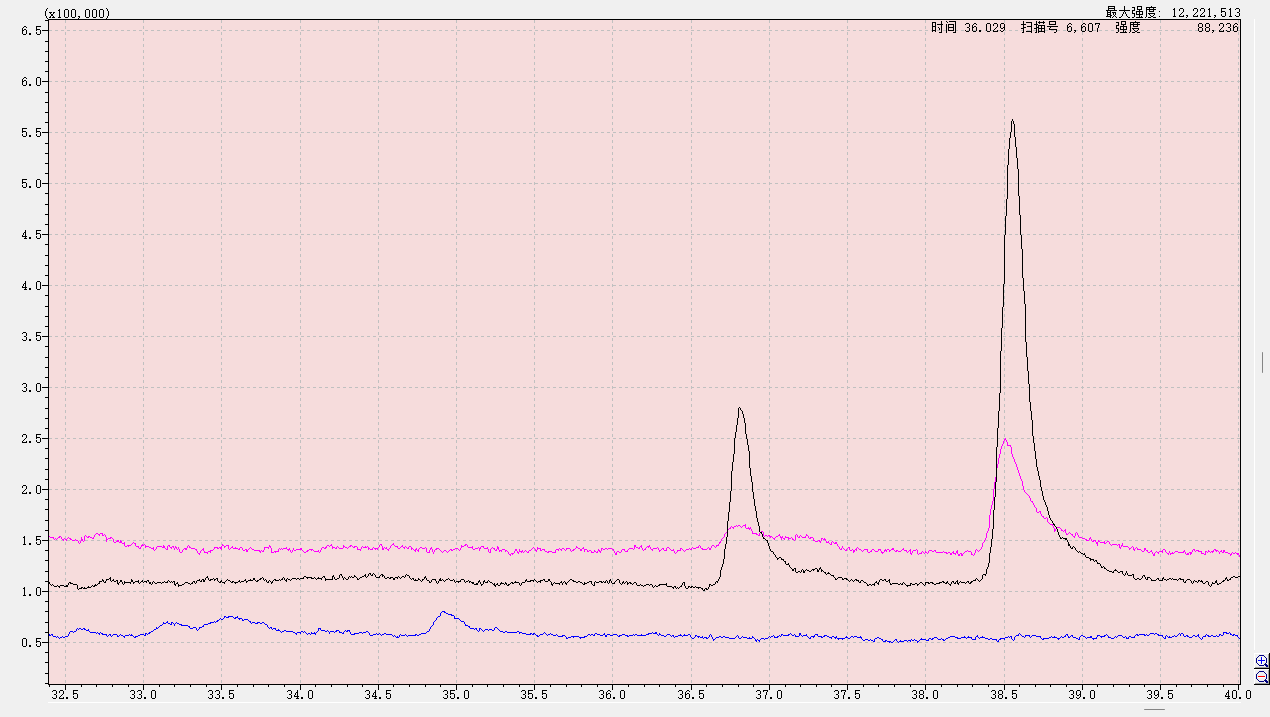


**Additional file 1: Figure S1.** GC/MS profiles of with Po1g ΔKU70 and Po1g KCrMAS. The pink, blue and black lines represent the solvent extracts of Po1g KCrMAS (engineered yeast) and Po1g ΔKU70 (control) cultures, and amyrin chemical standard, respectively.

**
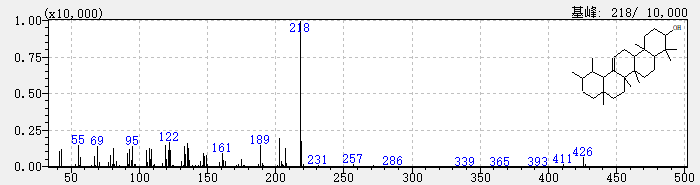
**

**A**

**
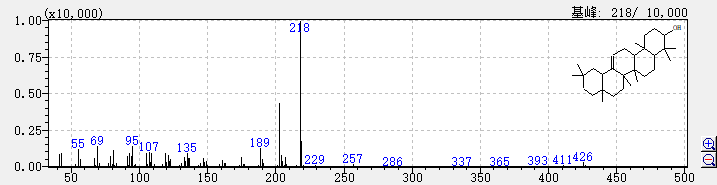
**

**B**

**
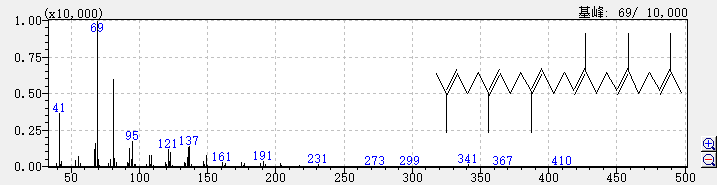
**

**C**

**Additional file 1: Figure S2.** Mass spectrum of amyrin and squalene chemical standards. A: α-amyrin; B: β-amyrin; C: squalene.





**Additional file 1: Figure S3.** Cell density of *Y. lipolytica* with (red circle) and without (black square) overexpression of CrMAS*.*


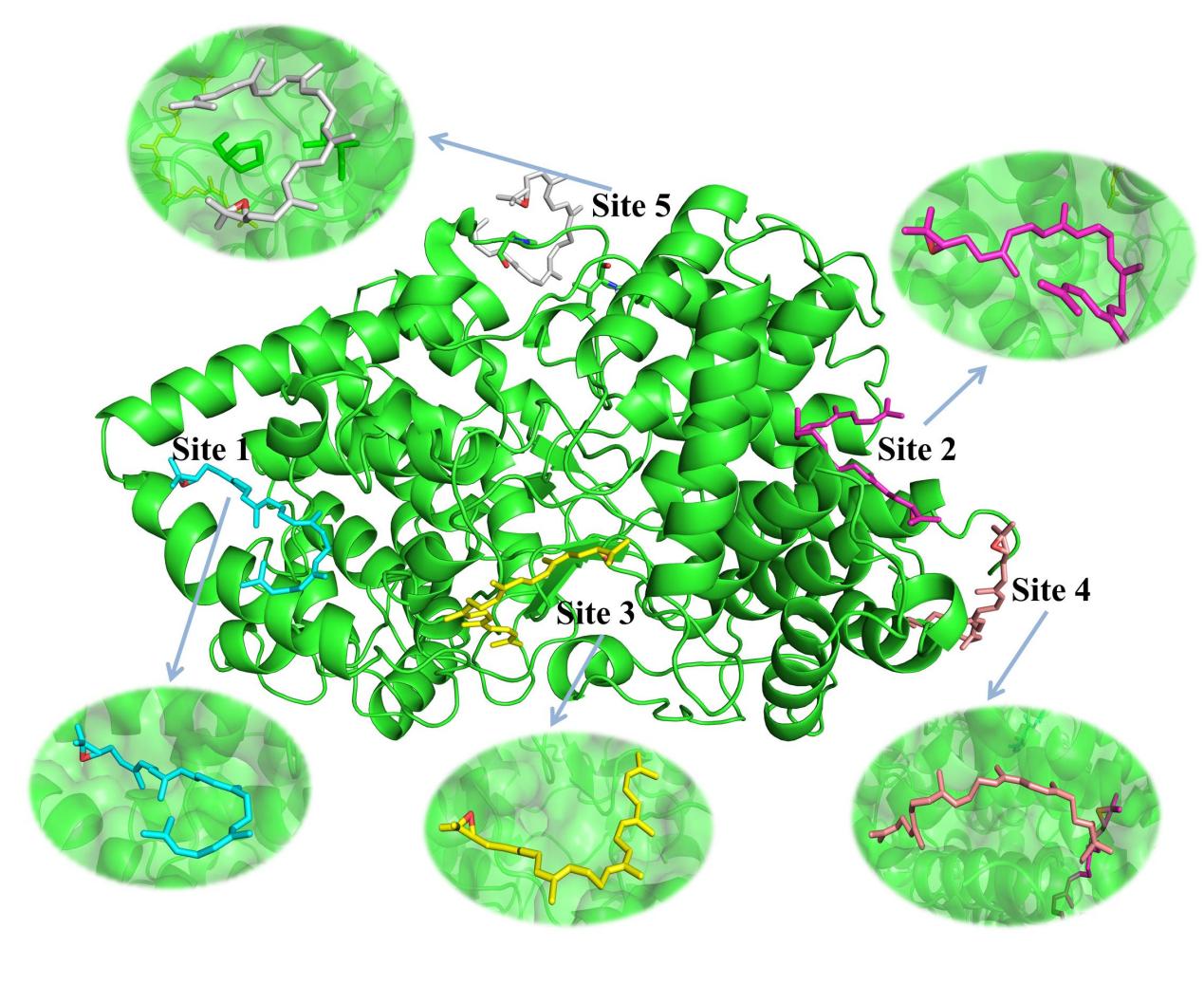


**Additional file 1: Figure S4.** Predicted ribbon model of CrMAS protein. Sites 1 to 5 represent the possible binding pockets of 2,3-oxidosqualene.


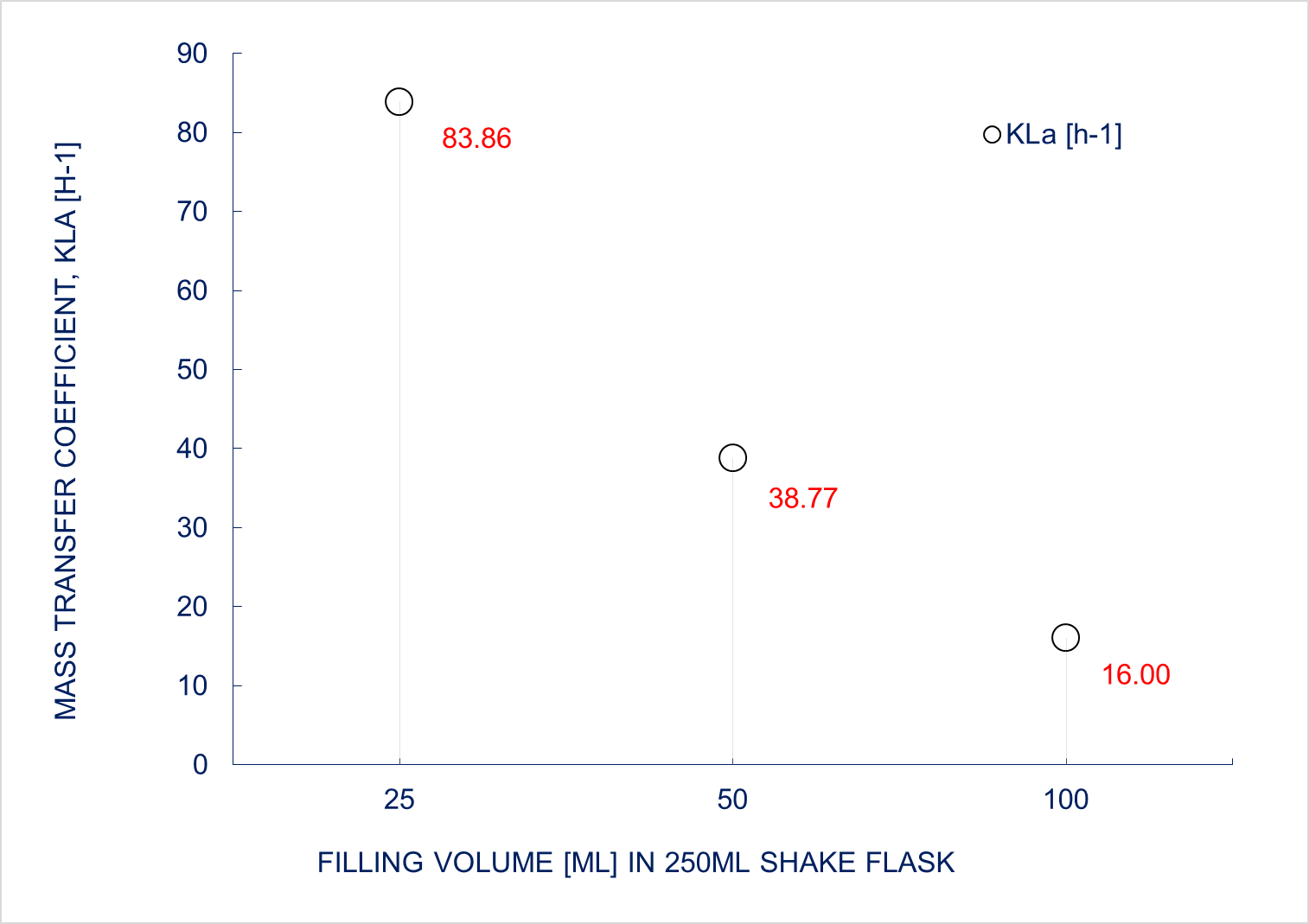


**Additional file 1: Figure S5.** Comparison of volumetric oxygen mass transfer coefficient to liquid filling volumes in 250 mL shake flask at 250 rpm.


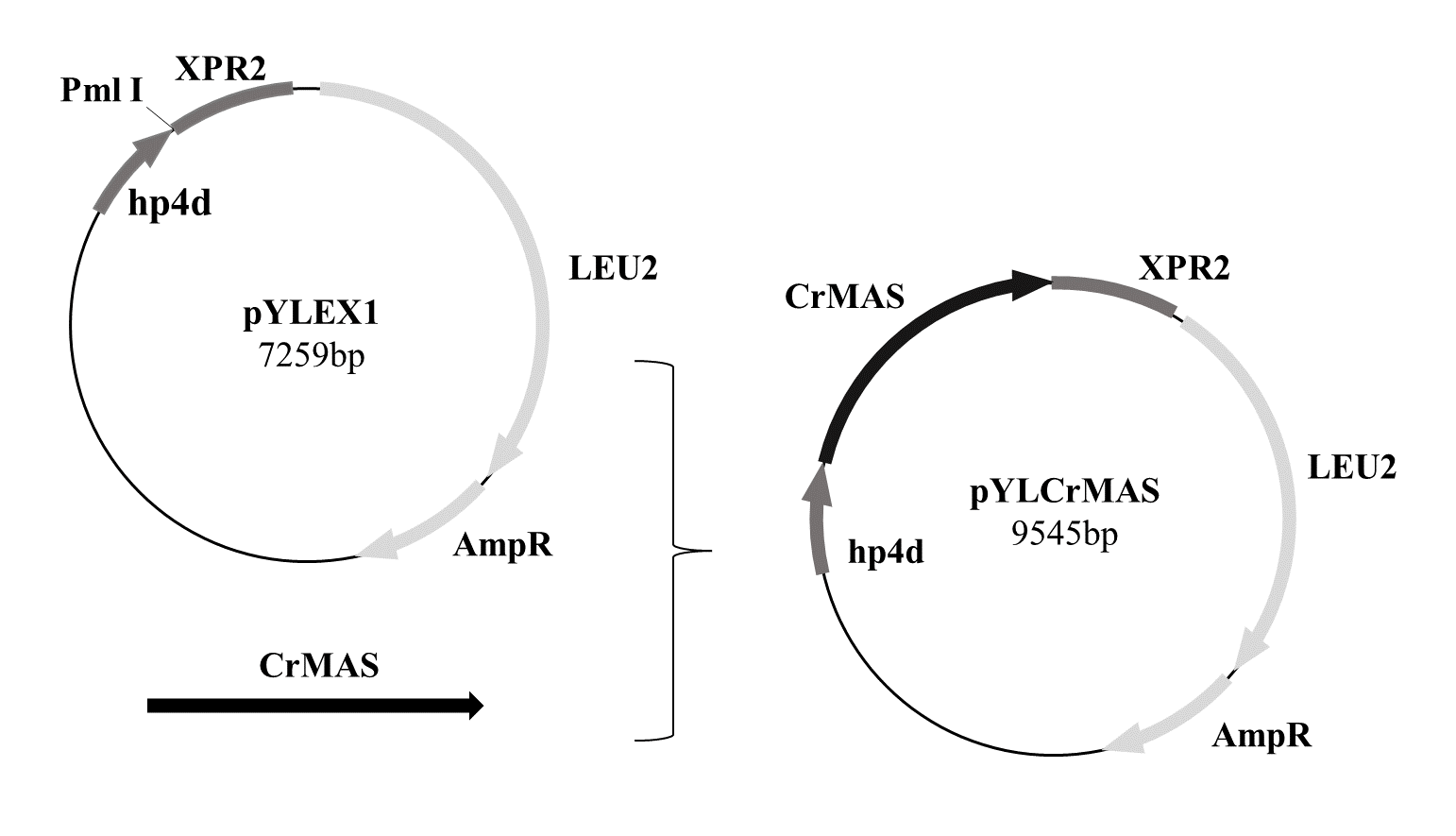


**Additional file 1: Figure S6.** Assembly of plasmid pYLCrMAS. *CrMAS* gene isolated from *Catharanthus roseus* was cloned into pYLEX1 with primer pair CrMAS-F/CrMAS-R to generate plasmid pYLCrMAS.

**ADDITIONAL FILE 1: METHODS**

**Preparation of *Y. lipolytica* Po1g** **△KU70 competent cell**

1. Inoculate a colony of *Y. lipolytica* Po1g △KU70 from a fresh YPD plate in 10 mL YPD medium (1% yeast extract, 2% peptone, 2% dextrose and 50 mM citrate buffer pH 4.0) in a 250 mL flask. Incubate with shaking at 225 rpm and 30°C for 20 hours.

2. Pellet the cells by centrifuging for 5 minutes at 5,000 g at 4°C.

3. Wash the cells with 20 mL TE buffer and pellet the cells as per Step 2.

4. Resuspend the cells in 1 mL of 0.1 M lithium acetate (pH 6.0, adjusted with acetic acid) and incubate for 10 minutes at room temperature.

5. Aliquot the competent cells (100 µL) into sterile 1.5 mL tubes. Proceed to the transformation steps below immediately or add glycerol to a final concentration of 25% (v/v) and store at -80°C for long-term storage.

**Transformation of *Y. lipolytica* Po1g △KU70**

1. Gently mix 10 µL of denatured salmon sperm DNA (10 mg/mL) and 1-5 µg of the linearized plasmid together with 100 µL of competent cells and incubate at 30°C for 15 minutes.

2. Add 700 µL of 40% PEG-4000 (dissolved in 0.1 M lithium acetate pH 6.0), mix well and incubate at 30°C for 60 minutes with shaking (225 rpm).

3. Heat shock the transformation mixture at 39°C for 60 minutes.

4. Add 1 mL YPD medium and recover for 2 hours at 30°C and 225 rpm.

5. Centrifuge at 10,000 g for 1 minute. Discard the supernatant and resuspend the cell pellet in 1 mL of sterile water.

6. Centrifuge the cells once more and discard the supernatant again.

7. Resuspend the washed pellet in 100 µL of sterile water and plate onto YNB selective plates (leucine-deficient plates).

**DNA sequence of the codon-optimized *CrMAS* gene (2286 bp)**

ATGTGGAAGCTGAAGATCGCCAAGGGCAAGGGCCCCTATCTGTATTCTACTAACAACTTCGTGGGCCGACAGATTTGGGAGTACGACCCCAATGCCGGCACTCCCCAAGAAAGAGAGGCTTTTGAAAAGGCCAGAGAGCAGTTCAGAAACAACCGAAAGAAGGGCGTGCACAACCCCTGCGCCGATCTGTTTATGCGAATGCAGCTGATCAAGGAGAACGGCATCGACCTGATGTCTATCCCCCCCGTGAGAGTGGAAGAGAAGGAGGAACTGACCTTCGAGAAGACCACCATCGCCGTGAAGAAGGCCCTGCGACTGAATCGAGCTATCCAGGCCACTGATGGCCACTGGCCTGCTGAAAATGCTGGACCTATGTTCTTTACTCCCCCCCTGCTGATCGCCCTGTACATCTCTGGAGCCATCAACACCATCCTGACCTCTGAGCACAAGAAGGAGCTGGTGCGATACATCTACAACCACCAGAACGAGGACGGCGGCTGGGGATTTTATATCGAGGGACATTCTACTATGATCGGCTCTGCCCTGTCTTACGTGGCCCTGAGACTGCTGGGAGAAGGCCCTGATGATGGAGATGGCGCTGTGGGAAGAGGACGACAGTGGATTCTGGATCACGGCGGCGCTACTGGTATTCCTTCTTGGGGAAAAACTTACCTGTCTGTGCTGGGCGTGTATGACTGGGACGGATGCAATCCTCTGCCTCCCGAGTTCTGGCTGTTTCCCTCTTTCTTCCCCTACCACCCCGCCAAGATGTGGTGCTATTGTCGAACCACCTACATGCCCATGTCTTACCTGTACGGCAAGAAGTACCACGGCCCCCTGACTCATCTGGTGATGCAACTGCGACAGGAGATCCACGTCAAGCCCTACGACCAGATCGACTGGAACAAGGCCCGACACGACTGCTGCAAAGACGACCTGTATTACCCCCACTCTTTCATCCAGGACCTGCTGTGGGACACCCTGAACTACTTCTCTGAGCCCGTGATGCGACGATGGCCCTGTAATAAGATCCGAGAGAAGGCCATGCGAAAGTGCATCAAGTACATGCGATACGGCGCCGAGGAGTCTCGATACATCACCATCGGCTGCGTGGAGAAGTCTCTGCAGATGATGTGCTGGTGCGCCCACGACCCTAATTGTGATGAGTTTAAGTACCACCTGGCCCGAGTGCCCGATTACCTGTGGCTGGCTGAAGATGGAATGAAGATGCAGTCTTTCGGCTCTCAGCTGTGGGACTGCACCCTGGCTACTCAGGCTATTATCGCTACCGGAATGGTGGAGGAGTACGGCGATACCATCAAGAAGGCCCACTTCTACATCAAGGAGTCTCAGGTGAAGGAGAACCCCAAGGAGGACTTCAAGGCCATGTACCGACACTTCACCAAGGGCTCTTGGACCTTCTCTGACCAGGACCAGGGCTGGGTGGTTTCTGATTGTACTGCTGAGGCTCTGAAATGTCTGCTGGTGTGCTCTCAGATGCCCCAGGACCTGGCTGGAGAAAAAGCCGATGTGGAACGACTGTACGATGCCGTGAACGTGCTGCTGTACCTGCAGTCTCCCGAGTCTGGCGGATTTGCCATTTGGGAGCCTCCTGTGCCTCAACCCTATCTGCAGGTGCTGAACCCCTCTGAGCTGTTCGCCGACATTGTGGTGGAGCAGGAACACGTCGAGAATACCGCCTCTATCGTGCAGGCCCTGGTGCTGTTCAAACGACTGCATCCCGGACACCGAGAGAAGGAGATTGAAGTGTCTGTGTCTAAGGCCGTGCGATTCCTGGAGGGCAGACAGTGGCCCGATGGATCTTGGTATGGATATTGGGGCATCTGCTTCCTGTACGGCACCATGTTCGTGCTGGGCGGCCTGACTGCTGCTGGAAAAACTTATAAAAACTCTGAGGCCATCCGAAAGGCCGTGAAGTTCTACCTGTCTACCCAGAACGAGGAGGGCGGCTGGGGAGAATGTCTGGAATCTTGTCCTTCTATGAAGTACATCCCCCTGGAGGGCAACCGAACCAACCTGGTGCAAACCTCTTGGGCCATGCTGGGCCTGATGTACGGCGGACAAGCTGAGAGAGATCCCACTCCTCTGCATAAAGCTGCCAAGCTGCTGATCAATGCCCAGATGGACGACGGCGATTTCCCCCAGCAAGAAATTACCGGCGTGTACATGAAAAACTGCATGCTGCACTACGCCGAGTACCGAAACATCTTCCCCCTGTGGGCCCTGGCCGAATATCGAAAAAGAGTGTGGCCCACCAAGGCCCTG
